# Assessing Understory Complexity in Beech-dominated Forests (*Fagus sylvatica* L.) in Central Europe—From Managed to Primary Forests

**DOI:** 10.3390/s19071684

**Published:** 2019-04-09

**Authors:** Katharina Willim, Melissa Stiers, Peter Annighöfer, Christian Ammer, Martin Ehbrecht, Myroslav Kabal, Jonas Stillhard, Dominik Seidel

**Affiliations:** 1 Faculty of Forest Sciences, Silviculture and Forest Ecology of the temperate Zones, University of Göttingen, Büsgenweg 1, 37077 Göttingen, Germany; melissa.stiers@forst.uni-goettingen.de (M.S.); Peter.Annighoefer@forst.uni-goettingen.de (P.A.); Christian.Ammer@forst.uni-goettingen.de (C.A.); martin.ehbrecht@forst.uni-goettingen.de (M.E.); dseidel@gwdg.de (D.S.); 2 Carpathian Biosphere Reserve, Laboratory of Forest Sciences, Krasne Pleso 77, 90600 Rakhiv, Ukraine; myroslawk@gmail.com; 3 Forest Resources and Management, WSL Swiss Federal Institute for Forest, Snow and Landscape Research, Zürcherstrasse 11, 8903 Birmensdorf, Switzerland; jonas.stillhard@wsl.ch

**Keywords:** understory structure, management intensity, regeneration, *Fagus sylvatica* L., primary forests, terrestrial laser scanning, Carpathian Biosphere Reserve

## Abstract

Understory vegetation influences several ecosystem services and functions of European beech (*Fagus sylvatica* L.) forests. Despite this knowledge on the importance of understory vegetation, it is still difficult to measure its three-dimensional characteristics in a quantitative manner. With the recent advancements in terrestrial laser scanning (TLS), we now have the means to analyze detailed spatial patterns of forests. Here, we present a new measure to quantify understory complexity. We tested the approach for different management types, ranging from traditionally and alternatively managed forests and national parks in Germany to primary forests of Eastern Europe and the Ukraine, as well as on an inventory site with more detailed understory reference data. The understory complexity index (UCI) was derived from point clouds from single scans and tested for its relationship with forest management and conventional inventory data. Our results show that advanced tree regeneration is a strong driver of the UCI. Furthermore, the newly developed index successfully measured understory complexity of differently managed beech stands and was able to distinguish scanning positions located on and away from skid-trails in managed stands. The approach enables a deeper understanding of the complexity of understory structures of forests and their drivers and dependents.

## 1. Introduction

The understory, i.e., “all vegetation growing under an overstory” [1], is an important functional and structural component of temperate forests. Understory vegetation influences ecosystem functions, such as nutrient cycling [2] or biodiversity of stands [3]. Moreover, it interacts with animal communities [4], e.g., by serving as a food source or shelter, and may impact the future composition of tree species [5]. The understory of primary temperate forests is characterized by the presence of advanced regeneration, vertical heterogeneity, and the development of secondary crowns by trees not yet reaching the overstory [6]. In the few remaining beech (*Fagus sylvatica* L.) primary forests in Europe, a high density of natural regeneration, as well as a multi-layered understory structure, has been observed [7,8,9].

Recent studies have shown that understory vegetation contributes notably to the stand structure of a forest. Ehbrecht et al. [10] observed an increase in the overall three-dimensional stand structural complexity due to the presence of dense understory vegetation, such as thickets with shelterwood trees. An essential part of modern silvicultural practices in Europe is to emulate complex structures as found in primary forests [11,12]. To do so, we need quantitative information on the different elements of the structure of primary forests to serve as a reference [12,13,14].

Previous research has mainly focused on single attributes such as shrub cover [15,16], shrub height [12,14,17], or understory richness [18]. Such understory attributes were used to emphasize the influence of understory vegetation on the overall structure of a forest. Hinsely et al. [19] found that understory “density” has a crucial impact on the provision of resources and habitats for birds in temperate forests of Europe. For example, Anderson and Meikle [20] revealed that an increase in the “presence” of understory vegetation had a positive effect on the relative abundance of mice (*Peromyscus leucopus*) in temperate deciduous forests. 

With the ecological and functional importance of understory structures in mind, a quantitative measure of understory complexity is needed. Here, we define understory complexity as an integrative measure of the three-dimensional, architectural, and spatial arrangement of all plant organs in the understory of a given forest at a given point in time. 

Recently, new options of measuring stand structural attributes by 3D reality capturing were successfully used to quantify the spatial arrangement of plant material in forests [10,21,22]. Brolly et al. [23] showed that terrestrial laser scanning (TLS) has the ability to quantify tree regeneration. However, the potential of using 3D data for an assessment of forest understory is so far little explored. In this study, we developed a new method to quantify the structural complexity of forest understories based on TLS. We hypothesized (i) that the understory complexity index is driven by young tree regeneration. By this, attributes that can be expected to be related to reductions of structural complexity, such as skid-trails, should be detectable. Furthermore, we hypothesize (ii) that beech-dominated forests of different management types differ in the complexity of understory structure.

## 2. Materials and Methods

### 2.1. Study Sites

**Figure 1:**
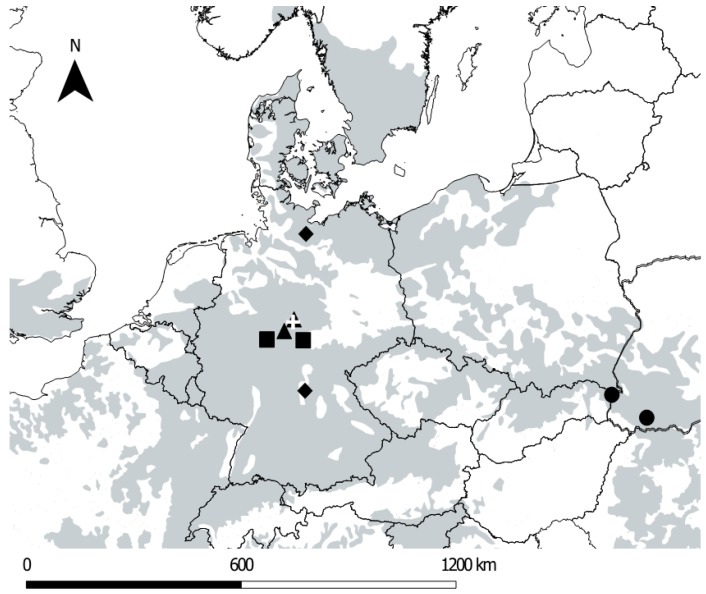
Distribution of *Fagus sylvatica* L. in Central Europe (grey area) and location of studied beech forests (▲= Traditionally managed, ♦ = Alternatively managed, ■ = National Parks, ● = Primary forests). Source of species distribution map: http://www.euforgen.org. The white + indicates the location of the inventory study site used for the young tree regeneration inventory (Waake, near Göttingen).

To address hypothesis (i), we used tree regeneration data from an inventory that was conducted in mixed broadleaf-coniferous even-aged stands in the region of Waake (administrative district of Göttingen, Lower Saxony, Germany). The site, from here on named “inventory site”, is located between 220 and 400 m above sea level (a.s.l.). To address hypothesis (ii), we investigated the understory in a series of beech-dominated forests across a gradient of management intensity, ranging from traditionally managed forests in Germany to primary forests in Slovakia and Ukraine [24]. A detailed description of the location of the study sites and important characteristics can be found in Figure 1 and Table 1. To ensure comparability, we determined certain selection criteria for our study plots at the sites. All plots were located in forest stands with a minimum beech share of 66% in the basal area. Furthermore, the last management intervention was dated back two or more years.

For each management type (traditionally managed, alternatively managed, national park [formerly managed forest], primary forest), we chose two geographical locations (Figure 1). Traditionally managed forests were selected in Lower Saxony State Forest in the forest districts of Hannoversch Münden and Reinhausen, respectively. Sites with alternative management (see explanation of ‘alternative’ below) were selected in the Northern German lowlands (Lübeck) and in the lower mountains of the Steigerwald, Bavaria (Ebrach), respectively. Two sites were placed in beech-dominated forests of the National Parks ‘Hainich’ and ‘Kellerwald-Edersee’. Finally, two sites were chosen from primary beech forests located in the Carpathian Mountains, where the largest remnants of primeval forests can be found in Europe [25]. We selected ‘Rožok’, a National Nature Reserve (NNR) in the Slovakian Republic and the primary forest Uholka-Shyroky Luh (Ukraine), which is a part of the Carpathian Biosphere Reserve (CBR) (Table 1). In addition to the site pairs according to the management type, a further site was used in Waake. This site was also considered to be traditionally managed, with the advantage that regeneration inventory data was also available for this site. 

Apart from the lowland sites in Lübeck (40–90 m a.s.l.), all study areas in Germany are located in the lower mountain ranges (190–635 m a.s.l.). The sites in the Western Carpathians were located highest, with 580–840 m a.s.l. The climatic conditions at all our study sites are considered temperate after the Köppen Geiger classification [26]. Annual mean temperature ranges from 6.5 °C to 8.5 °C, and annual precipitation varies between 600 and 1407 mm.

The four different management intensities correspond to four different management types that represent a gradient in management intensity (Table 2). Management in traditionally managed forests sites Reinhausen and Hannoversch Münden is based on the “Guidelines of beech forest management in Lower Saxony, Germany” [27]. These forests are characterized by a thinning cycle of 5 to 10 years during which up to three competitors per target tree are removed. Target trees are harvested when they have reached a target diameter of at least 65 cm at breast height (1.3 m). In the alternatively managed forest sites, the thinning frequencies and intensities are lower, so thinning cycles are longer, and less competitors are removed during the rotation period. The share of trees growing beyond the mentioned target diameter is also larger. In Lübeck, a further objective is to increase the growing stock by ceasing silvicultural activities within stand ages of 30–80 years. Finally, the forest districts Ebrach and Lübeck aim for a higher amount of coarse woody debris compared to Reinhausen and Hannoversch Münden. 

No management for at least two decades characterizes the sites in the National Parks Hainich and Kellerwald-Edersee, while the primary forests Uholka and Rožok have developed without a forest management concept [13].

To allow an appropriate comparison of the differently managed systems, we took the different age classes occurring in managed stands into account. These ranged from thickets (0–20 years) with shelterwood trees to mature timber stands (81–120 years) (Table 1). The mature stands in National Park Hainich had an average age of 180 years, which is comparable to the range of mean ages reported for the stands in the National Park Kellerwald-Edersee (174–194 years). The primary beech forests in Uholka and Rožok can be described as uneven-aged stands [9,28], which are mainly characterized by continuous, small-scale regeneration processes [9,29,30]. However, the average age of the mature trees in Rožok was 180–230 years [30], whereas the mean age of mature trees in Uholka was estimated to be 350 years [9]. For details on the primary beech forests of Rožok and Uholka, the interested reader is referred to Kucbel et al. [28] and Commarmot et al. [7].

### 2.2. Terrestrial Laser Scanning and Sampling Design

A Faro Focus 3D 120 Terrestrial Laser Scanner (Faro Technologies Inc., Lake Mary, FL, USA) was used on all sites. The instruments provides data with a ranging error of ±2 mm and a range noise between 0.3 and 2.2 mm depending on the reflectivity of the objects. All scans were conducted during dry weather conditions and with wind speeds below 10 ms^−1^. The scanner was always aligned horizontally (<5°) using its internal electronic level. The scanner was mounted on a standard tripod at breast height (1.3 m), ensuring enough space for the scanner to operate (0.6 m of clearance in all directions during scanning). For a field of view of 300° (vertically) × 360° (horizontally), an angular resolution of 0.035° was used during scanning, resulting in ~44.4 million measurements per scan. During the scans, the scanner’s standard filters (Clear Contour- and Clear Sky-filter) were applied.

In the region of Waake, we used 71 sample points from an inventory of tree regeneration, which were located on a systematic grid (100 m × 100 m) covering a total of 171.4 hectares. At each sample point of the inventory site, all juvenile trees (≥ 1.30 m height, < 7 cm diameter at breast height (DBH)) located inside the circular 10 m² plot area were counted. We conducted one single scan between October and November 2017 at the center of each of 71 plots. Trees within the plots were still partially foliaged.

**Figure 2:**
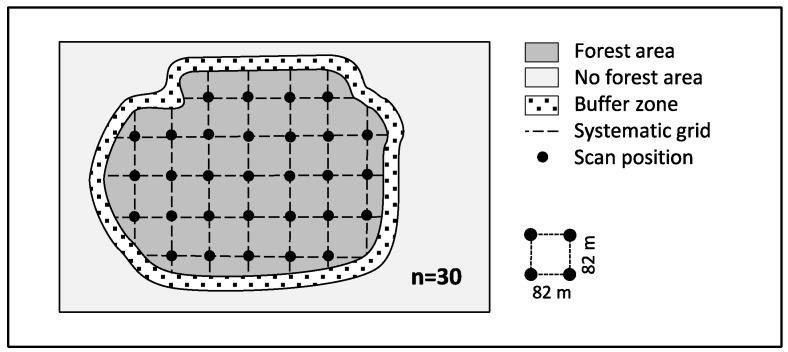
Sampling design for an exemplary plot (Forest area = ~20 ha).

**Figure 3:**
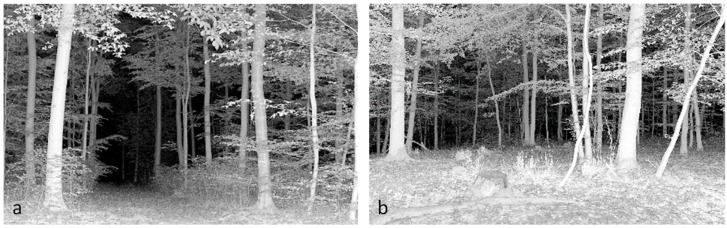
Exemplary locations of measurements on skid-trails (**a**) and away from skid-trails (**b**).

For all other sites (4 management types × 2 sites), we scanned at 30 sample points on a systematic grid (82 m × 82 m) in an area of about 20 hectares each (Figure 2). These scans were conducted between May and September 2017. A buffer-distance of 20 m to neighboring forest stands, forest edges, and roads was respected during scanning to avoid edge effects. For all managed stands, we recorded scans located on skid-trails or away from skid-trails (Figure 3).

### 2.3. Construction of An Understory Complexity Index

Each of the 30 single scans per plot generated a three-dimensional point cloud representing all detected hits in the vicinity of the scanner (120 m range) as xyz-coordinates. Each scan was imported to Faro Scene^®^ Software (Faro Technologies Inc., Lake Marry, FL, USA) and subsequently filtered using the software’s standard filter (Dark Scan Points, Outlier) as recommended by the manufacturer. In a last step, the point cloud of each scan was exported as xyz-file (Cartesian coordinates).

Each point cloud in xyz format was than imported to Mathematica^®^ software (Wolfram Research, Champaign, IL, USA) to compute the understory complexity index, from here on called UCI, based on a newly developed algorithm described as follows. First, in order to limit the extent of the analysis to the area in the proximity of the measurement site, we reduced the point clouds to hits within 15 m horizontal distance of the scanner. This should also reduce the effects of shadowing, which increase with distance. Then, to normalize the spatial density of the raw data, we homogenized the point cloud resolution further by using voxels (volumetric pixel) with an edge length of 1 cm. Voxeled point clouds are also less prone to measurement errors like, for example, beam divergence [31,32]. To account for uneven terrain, we then calculated digital terrain models from each scan using the lowermost hits in a 10 cm xy-resolution of the initial point cloud, keeping the original 1 cm resolution for the z-values (height). Based on the lowermost hit at each ‘xy-cell’, we interpolated the digital terrain model to the 1 cm resolution of the voxeled point cloud. Using the ground-level height from the digital terrain model, we then calculated normalized heights of each voxel by correcting it with the terrain level height at the xy-position of the voxel.

As the UCI was intended to describe the understory, we selected all voxels located between 0.8 and 1.8 m in height (0.5 m below and above the scanner). We decided to use the lower boundary of 0.8 m for this ‘layer’ as we wanted to exclude larger herb and shrub layer vegetation, which is most dominant below 0.8 m. We also wanted to reduce influences of lying deadwood on the data. The upper boundary of 1.8 m was chosen to have as little crown material from overstory trees as possible affecting the data.

All points of the resulting horizontal ‘slice’ were projected onto a horizontal plane. To do so, the height values (z-value) were set to zero (vertical projection). Then, the x- and y- coordinates were transformed into polar coordinates and sorted according to their azimuth angle using a resolution of 1° for further standardization. During this step, only the first hit in each direction was used for further processing. We then reconverted the polar coordinates to Cartesian coordinates, which were finally used to generate a polygon connecting all points. 

Based on the formula introduced by McGarigal and Marks [33], the fractal dimension index (FRAC) for the polygon of each single-scan was calculated:

FRAC = (2 × ln (0.25 × P))/ln (A)(1)

with ln being the natural logarithm to the base e, P being the perimeter, and A the area of the polygon (see also [10]). 

FRAC is as a measure of shape complexity [33] and we used it to characterize the degree of complexity of the polygons, each representing hits within the horizontal cross-section through the stand, as visible from the specific location of the scanner. This FRAC-value is the final result of the processing chain of the UCI.

**Figure 4:**
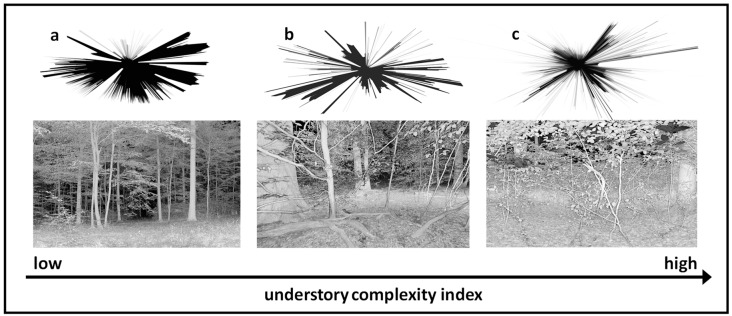
Exemplary horizontal cross-sectional polygons and corresponding images of stands with a low (**a**); intermediate (**b**), and high understory complexity index (UCI) value (**c**).

In its construction, the UCI makes use of a similar approach as the stand structural complexity index (SSCI) introduced by Ehbrecht et al. [10]. While the SSCI used multiple vertical cross-sections through a single scan point cloud (see Ehbrecht et al. [10] for further detail on the method), we used a single horizontal cross-section to derive the UCI as explained above (Figure 4). We argue that due to its construction, the UCI increases with increasing number and distributional irregularity of plant objects in the understory. We determined the UCI for all scans made in the eight study sites as well as on the site where we conducted the young tree inventory.

### 2.4. Statistics

The statistical analyses were conducted with the software environment R, version 3.3.3 (R Development Core Team 2017). To determine differences in understory complexity depending on the presence of tree regeneration, skid-trails, and for different management types and study areas, we used the non-parametric Kruskal–Wallis test, because normal distribution and homogeneity of variance could not be assumed. For post-hoc analysis, we used the Wilcoxon rank sum test with Bonferroni corrected *p*-value. Both tests were conducted at the alpha-level of 0.05.

## 3. Results

### 3.1. Effect of Tree Regeneration and Skid-Trails on the UCI

**Figure 5:**
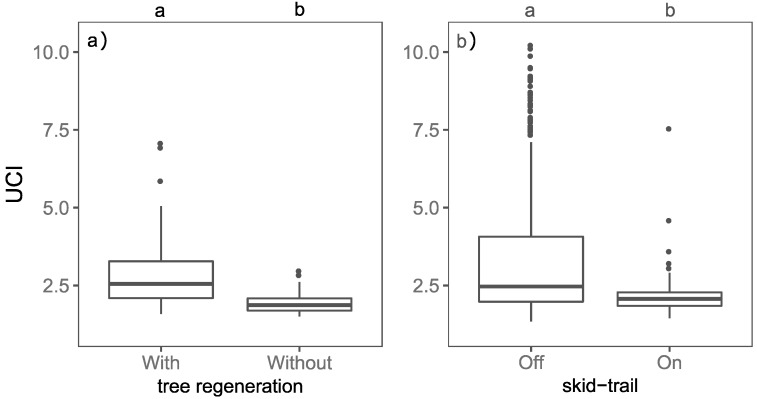
(**a**) Box-and-whisker plots of the understory complexity index (UCI) in dependence of the presence of tree regeneration in the inventory plots. The letters a and b (*p* < 0.05) indicate significant differences between samples with and without tree regeneration. Sample sizes: with tree regeneration (n = 27) and without tree regeneration (n = 44). (**b**) Box-and-whisker plots of the understory complexity index (UCI) in dependence of the presence of skid-trails in the managed beech stands. The letters a and b (*p* < 0.05) indicate significant differences between samples in which we measured on skid-trails and off skid-trails. Sample sizes: on skid-trails (n = 54) and off skid-trails (465).

The dataset of tree regeneration was used to verify the performance of the UCI with regard to the presence of understory regeneration. The scan locations with tree regeneration (Figure 5a), have on average a significant higher UCI (a) than the scan locations without tree regeneration (b). In the managed beech stands (Figure 5b), the scan locations on skid-trails (b) have a significantly lower UCI than the samples taken off skid-trails (a).

### 3.2. UCI of Beech Stands with Regard to Different Management Types

**Figure 6:**
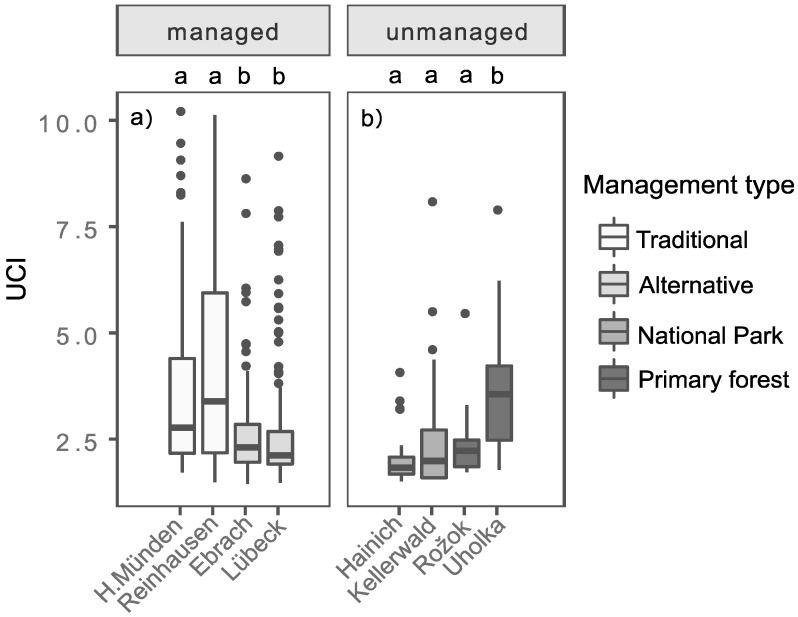
(**a**) Box-and-whisker plots showing the understory complexity index (UCI) values for all managed study sites. The letters a and b indicate significant differences between the traditionally managed and the alternatively managed study sites at *p* < 0.05. Sample size for managed study sites: Hann. Münden (n = 120), Reinhausen (n = 120), Ebrach (n = 120), and Lübeck (n = 120). (**b**) Box-and-whisker plots showing the understory complexity index (UCI) values for all unmanaged study sites. The letters a and b indicate significant differences between the study sites of the National parks and the primary forests at *p* < 0.05. Sample size for unmanaged study sites: Hainich (n = 30), Kellerwald (n = 30), Rožok (n = 30), and Uholka (n = 30). Different colors represent the different management types.

The UCI differed for the investigated managed (Figure 6a) and unmanaged (Figure 6b) beech stands. With focus on the managed study sites (Figure 6a), the UCI of the traditionally managed forest sites (Hann. Münden = 2.77, Reinhausen = 3.39) is on average significantly higher (a) than the UCI of the alternatively managed forest sites (Ebrach = 2.30, Lübeck = 2.12). Within the management type “Traditional” and ”Alternative”, no significant difference in the UCI could be found between the study locations, respectively (Hann. Münden = Reinhausen and Ebrach = Lübeck). Considering the different age classes of the managed forests (Table 3), we observed on average a decreasing trend of the UCI from thickets with mature overstory trees (0–20) to immature timber stands (41–80) in traditionally managed forest sites and mature timber stands (81–120) in alternatively managed forest sites. Looking at the UCI of the unmanaged study sites (Figure 6b), there is an increasing trend from the National Parks Hainich (1.83) and Kellerwald (1.98) over the primary forests Rožok (2.22) to Uholka (3.56), even though only Uholka differed significantly (b) from the other unmanaged sites (Hainich = Kellerwald = Rožok < Uholka). Within the unmanaged forest sites, Uholka had the highest UCI. 

## 4. Discussion

### 4.1. Important Drivers of Understory Complexity

Prior studies have shown a relationship between understory density or understory diversity and biodiversity [15,34,35]. This indicates the importance of the understorey structure and its precise and objective description based on quantitative information. We introduced the UCI to allow for an objective, solely mathematically way of assessing understory complexity. When interpreting the results, one has to consider that in our study, the mean UCI of all plot-based single scans describe understory complexity on stand scale (α-level) only.

Our results showed that there is a significantly higher UCI (a) on our inventory plots with tree regeneration compared to the inventory plots without regeneration (Figure 5a). This finding indicates that regeneration is a strong driver of the UCI (hypothesis (i)). The UCI also proved to be sensitive to skid-trails. The UCI of samples measured on skid-trails is significantly lower (b) compared to samples measured off skid-trails (Figure 5b). Skid-trails are usually cleared of regeneration and other vegetation and therefore show lower UCI values.

The observed importance of tree regeneration for the UCI is consistent with the findings of Ehbrecht et al. [10], who have already observed that the structural complexity of entire stands increases with overall tree density (trees with DBH > 7 cm). Consequently, an increase in the density of plants seems to have an increasing effect on both overall stand and understory structural complexity. Despite the higher UCI values with the presence of regeneration, it is worth noting that our data also showed a certain variation in UCI values measured on skid-trails with no juvenile tree present (Figure 5b). Thus, as one would expect, tree regeneration is not the only driver of UCI. In general, according to McElhinny et al. [36], structural complexity “involves the interaction between a number of different attributes”. Therefore, it is likely that in addition to regeneration density, the UCI is driven by other understory attributes, like understory species richness [18], shrub height [15], or architecture, as well as the overall diameter distribution of the trees.

### 4.2. Effects of Different Management Intensities on the UCI

Our study showed that UCI differed for different management types, so hypothesis (ii) could be confirmed. In Central Europe, thinning is an essential part of forest management [37], resulting in important structural differences during forest development. Accordingly, thinning and harvest frequencies, timing, and intensities varied in the investigated beech plots and are likely the main causes of the observed differences. In our study, the highest UCI was found in traditionally managed even-aged thickets with mature overstory trees (Table 3). To initiate this developmental phase, about 30% of the growing stock was harvested in one intervention [38] and the remaining overstory trees have continuously been removed over the last 30 years.

In the stands between 21 and 40 years, the branch-free section of the stems became more dominant, and the tree crowns, which generally increase the complexity due to the presence of tiny irregular structures such as leaves and twigs, were only partly located in the layer that was considered for the UCI. The effect of the absence of crown elements on the UCI can be seen in the immature timber stands (age class ‘41–80’). Here, stems are mainly branchless in the height layer relevant to determining the UCI (0.8–1.8 m).

During the last decades, single tree selection (target diameter harvest) as a regeneration form has gained increasing importance in traditional forest management [38]. This approach ultimately results in small uneven-aged regeneration patches across the whole forest stand as opposed to shelterwood systems aimed at regenerating an expanded area homogeneously and even-agedly. In any case, the beech stands between 81 and 120 years considered here are mainly being regenerated through single tree selection whereby many of these stands were not being regenerated thoroughly yet, which explains why the UCI was still rather low in this age class.

Reduced thinning and harvesting frequencies and intensities, as conducted in beech stands referred to as alternative management type here, also resulted in significantly lower UCI values (Figure 6a; Table 3). Presumably, this can also be explained by the higher canopy densities, resulting in a weaker development of the understory due to the lack of light. The effects of varying canopy densities on the growth of saplings is well documented [39,40,41]. 

In the investigated unmanaged forest sites, we found an increasing trend in UCI from the National Parks to the primary forests, even if we could not find significant differences between the UCI of both National Parks and the primary forest Rožok (Figure 6b). The comparatively low UCI values for the National Parks can be explained by the fact that management was ceased in these forests at the peak of what can be referred to as a ’vault-like’ forest structure (German: “Hallenwälder”), characterizing the optimal phase of beech forests when canopy densities are high and trees are still comparably vital. Therefore in this stage, the beech stands of both National Parks were characterized by a single canopy layer and pronounced shade in the understory (canopy cover regularly higher than 90%; for example, see Ref. [42]). In the year of measurement, this condition was still relatively pronounced at both study sites in the National Parks due to a low age-induced mortality and the absence of high severity disturbance events [43]. Therefore, a comprehensive regeneration layer could not develop yet [14,44,45], which was represented by the low UCI values found here. 

Interestingly, our results showed significant differences between the UCI of both primary forests. Gaps from the senescence of single trees or small tree groups are the most prominent initiators of regeneration in primary European beech forests [9,29,30,46]. With an estimated mean age of ~220 years of the mature trees, the sampled stand in Rožok appeared to still be in the transition from the optimum phase to senescence and the decay phase [24]. The general absence of gaps was presumably also the reason here and for why a pronounced understory was missing in the parts of the Rožok primary forest we sampled, which again resulted in a low UCI. Compared to Rožok, the sampled stands in the primary forest Uholka were comprised of a higher average age of the overstory trees and showed a significantly higher UCI (Figure 6b; Table 3). Apparently, the decay phase was more advanced in these stands, as characterized by a higher abundance of canopy gaps and corresponding understory development compared to Rožok [47]. As a result of several small and medium sized gaps [8,48,49], earlier studies detected large regeneration stocks [12], which is also reflected in our higher UCI values. However, not only is the density of natural regeneration a crucial driver of UCI, the heterogeneity of different heights of the saplings and young trees is as well. Due to continuous, small-scale disturbance events [9], in several European primary beech forests, different age classes of natural regeneration coexist [7,12,14,17]. This resulted in heterogeneous understory structures as observed in virgin beech forests in Uholka [14] and may also contribute to the high complexity of the understory measured by the UCI. 

Generally, understory development is not only a question of age but also of browsing pressure through ungulates like roe deer and red deer [40]. At least for Uholka, Hobi et al. [29] recorded low browsing damage. For Rožok, no recent information is available. However, varying ungulate densities can also affect the understory structure and, with this, the values of our UCI.

## 5. Conclusions

The aim of the presented study was to examine the possibilities in recording and measuring the complexity of understory forest structure objectively and efficiently using terrestrial laser scans. The suggested understory complexity index (UCI) has proven to yield plausible results distinguishing a variety of stand situations and allows quantitatively comparing these with one another, as presented here for beech forests. 

Because of the fact that we found significantly lower UCI values on scan locations without tree regeneration than on scan locations with tree regeneration, we conclude that the presence of young, established trees is a strong driver for the UCI. The significantly lower UCI values measured on skid-trails without vegetation around the scanner position support this assumption.

The observed significant differences in the UCI between traditionally and alternatively managed stands and between the national parks and the primary forest Uholka show that the UCI is able to distinguish the understory structure of differently managed forest sites. In addition, the significant differences between the different age classes show that the UCI is capable of differentiating understory complexity of the investigated even-aged stands. These results support empirical findings. In the investigated managed beech-dominated stands, the structural complexity of the understory is either large during the early phases of stand development (even-aged thickets with mature overstory trees; see age class 0–20) or when the senescence of trees has largely proceeded, as in the primary forest Uholka (age class ~350), initiating the understory development. 

Analyzing the influence of additional understory-related attributes on UCI, such as the presence of large shrubs, effects of understory species richness, or the diameter variability of the overstory trees may be an important future research task, next to the comparison of forests dominated by different tree species (e.g., conifer forest vs. deciduous forest; early successional vs. late successional forests).

## Figures and Tables

**Table 1:** Information on location and basic environmental conditions of the study sites. For each management type, we chose two study sites. For each study site of the management type “Traditional” and “Alternative”, the age classes “0–20”, “21–40”, “41–80”, and “81–120” were considered.

Country	Management Type	Study Sites	Mean Temperature (°C)	Precipitation (mm y^−1^)	Elevation (m a.s.l.)	Age Class (Years)
**Germany**	Traditional	Waake (inventory site)	7.5	750	220–400	0–20, 21–40, 41–80, 81–120, 121–190
**Germany**	Traditional	Hann. Münden Reinhausen	6.5–7.5 8	750–1050 740	270–410 190–310	0–20, 21–40, 41–80, 81–120
	Alternative	Ebrach Lübeck	7–8 8–8.5	850 625–725	320–480 40–90	0–20, 21–40, 41–80, 81–120
	National Park	Kellerwald Hainich	6–8 7–8	600–800 600–800	540–635 330–380	~180 ~180
**Slovakia**	Primary forest	Rožok	6–7	780	580–745	~220
**Ukraine**		Uholka	7	1407	700–840	~350

**Table 2:** Degree of intervention for traditionally managed and alternatively managed beech forests, National Parks, and primary forests.

Management Type	Degree of Intervention
Traditional	Yield-orientated with thinning cycles of 5 to 10 years and removal of up to 3 competitors per intervention; target-diameter harvest
Alternative	Compared to traditional forestry, lower thinning and harvesting frequencies and intensities + additional management goals
National Park	Unmanaged for 20–30 years
Primary forest	Unmanaged, no or minimal human impact

**Table 3:** Descriptive statistics of understory complexity index (UCI) for each management type and age class. Different letters indicate significant differences between the age classes at *p* < 0.05.

Management Type	Age Class	Mean	Median	Minimum	Maximum	Standard Deviation	Variance	Coefficient of Variation (%)
Traditional	all	3.89	2.91	1.49	10.23	2.24	5.02	57.53
	0–20 **^e^**	6.05	6.47	1.76	10.23	2.51	6.31	41.54
	21–40 **^a^**	4.06	3.93	2.11	8.67	1.62	2.63	39.88
	41–80 **^bc^**	2.25	2.10	1.67	7.64	0.77	0.59	34.17
	81–120 **^ba^**	3.25	2.47	1.49	9.52	1.73	2.99	53.30
Alternative	all	2.67	2.22	1.44	9.18	1.35	1.82	50.40
	0–20 **^a^**	4.18	3.73	1.91	9.18	1.85	3.42	44.25
	21–40 **^b^**	2.34	2.31	1.78	3.41	0.33	0.11	14.16
	41–80 **^cd^**	2.13	2.02	1.48	3.52	0.41	0.17	19.24
	81–120 **^cd^**	2.05	1.84	1.44	6.10	0.74	0.55	36.07
National Park	~180 **^cd^**	2.26	1.86	1.34	8.11	1.15	1.33	50.93
Primary Forest	all	2.96	2.47	1.72	7.92	1.31	1.71	44.18
	~220 **^bcd^**	2.34	2.22	1.72	5.48	0.73	0.53	31.19
	~350 **^a^**	3.60	3.56	1.77	7.92	1.47	2.16	40.78
